# Cracking the Code of a Rare Colonic Mesentery Gastrointestinal Neuroendocrine Tumor Disguised as Chronic Gastroenteritis: The Critical Art of Timely Detection

**DOI:** 10.7759/cureus.45465

**Published:** 2023-09-18

**Authors:** Colleen Achong, Wai Lin Thein, Widz Y Douillard, Madhumati Kalavar, Kirwin Gibbs

**Affiliations:** 1 Medicine, One Brooklyn Health, Brooklyn, USA; 2 Internal Medicine, One Brooklyn Health, Brooklyn, USA; 3 Hematology and Oncology, One Brooklyn Health Interfaith Medical Center, Brooklyn, USA; 4 Radiology, One Brooklyn Health, Brooklyn, USA

**Keywords:** carcinoid tumor, rare tumors, primary site neuroendocrine tumor, mesenteric mass, neuroendocrine neoplasm

## Abstract

Neuroendocrine tumors (NETs) typically present in the setting of metastasis from other solid organs and are considered late manifestations of the disease. Therefore, primary tumors are extremely rare. NETs of the colonic mesentery occur more than 70% of the time in the appendix, small intestine, and rectum. Here, we describe the case of a patient who presented with multiple episodes of diarrhea and abdominal pain, which was waxing and waning in occurrence, with CT findings of a rare primary NET.

## Introduction

Neuroendocrine tumors (NETs) are rare heterogeneous tumors with an incidence ranging from one to five per 100,000 patients, arising from the neural crest cells (Kulchitsky cells), enterochromaffin cells, and enterochromaffin-like cells, and have both neural and endocrine components [1]. Malignancy is not infrequent, and when malignant, NET is diagnosed at advanced stages with distant metastases because of its indolent nature. The most common sites are gastroenteropancreatic and tracheobronchopulmonary. Other sites include the parathyroid, adrenal, and pituitary glands; calcitonin-producing C-cells of the thyroid; and less common sites such as the skin and soft tissues [1]. NET cells release tryptophan, serotonin, monoamine oxidase, and other substances. These bioactive substances cause patients to present with symptoms such as flushing, diarrhea, dyspnea, palpitation, and bronchospasm. Some complications include carcinoid heart, mesenteric fibrosis pellagra, neuropsychological disorders, and carcinoid crisis.

## Case presentation

An 85-year-old female with a past medical history of chronic normocytic normochromic anemia gastrointestinal bleeding due to chronic non-steroidal anti-inflammatory drug (NSAID) use for osteoarthritis, hypertension, hyperlipidemia, grade 2 diastolic heart failure with bilateral lower extremity edema, cataract, and depression initially came to the emergency department (ED) with complaints of headache, generalized body pain more in the epigastric region, and ongoing diarrhea for five days. These episodes had started approximately five months ago, occurred every three to four weeks, and resolved in two to three days. This time it was unusual that it lasted for five days. Because she felt very tired, she decided to visit the hospital. The patient had refused endoscopy and colonoscopy despite multiple referrals and frequent medical education on the necessity.

In the ED, the patient was given acetaminophen, famotidine, loperamide, ondansetron, and intravenous hydration with normal saline.

Vitals on presentation were blood pressure of 149/84 mmHg, heart rate of 72 beats/minute, respiratory rate of 20 breaths/minute, oxygen saturation of 94% on room air, and temperature of 97.9°F. Lab results on admission are presented in Table 1.

**Table 1:** Laboratory findings on admission. WBC: white blood cells; Hb: hemoglobin; MCV: mean corpuscular volume; BUN: blood urea nitrogen: eGFR: estimated glomerular filtration rate; Na: sodium; K: potassium; CO_2_: bicarbonate; ALT: alanine aminotransferase; AST: aspartate aminotransferase; ALP: alkaline phosphatase; Ca: calcium; BNP: brain natriuretic peptide; PT: prothrombin time; INR: international normalized ratio; PTT: partial thromboplastin time; HbA1C: glycated hemoglobin

Test	Values	Reference range and units
WBC	13.4	4.5–11.0 × 10^3^/µL
Hb	11.4	11.0–15.0 g/dL
MCV	87.9	80–100 fL
Platelets	339	130–400 × 10^3^/µL
BUN	22	7.0–18.7 mg/dL
Creatinine	2.2	0.57–1.11 mg/dL
eGFR	21.4	≥90.0
Na	138	136–145 mmol/L
K	5.1	3.5–5.1 mmol/L
CO_2_	16	22–29 mmol/L
Anion gap	13	4 to 12 mmol/L
Total bilirubin	0.5	0.2–1.2 mg/dL
ALT	17	10–55 U/L
AST	33	5–34 U/L
ALP	67	40–150 U/L
Albumin	3.6	3.5–5.2 g/dL
Phosphorous	3	2.3–4.7 mg/dl
Ca	8.3	8.4–10.2 mg/dL
BNP	395	10–100 pg/ml
Lactate	1.2	0.50–1.90 mmol/L
High-sensitivity troponin	70.5	0–17 ng/mL
PT	9.7	9.8–13.4 seconds
INR	0.82	0.85–1.15
PTT	27	24.9–35.9 seconds
HbA1c	5.9	4.8–5.6%
Blood culture	Negative	Negative

**Figure 1:**
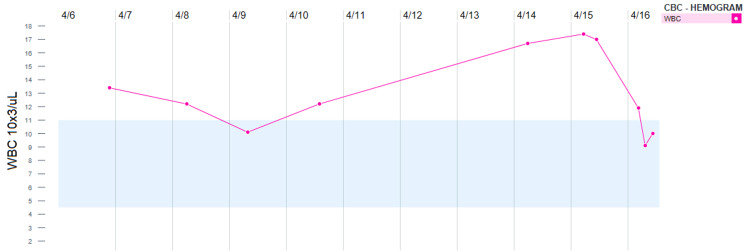
Leukocytosis trend. CBC: complete blood count; WBC: white blood cell

Due to leukocytosis and diarrhea, infectious pathology was suspected. Metronidazole was started on admission. Later, she received one dose of doxycycline and vancomycin orally due to the re-occurrence of diarrhea. The trend of leukocytosis is illustrated in Figure 1.

**Figure 2:**
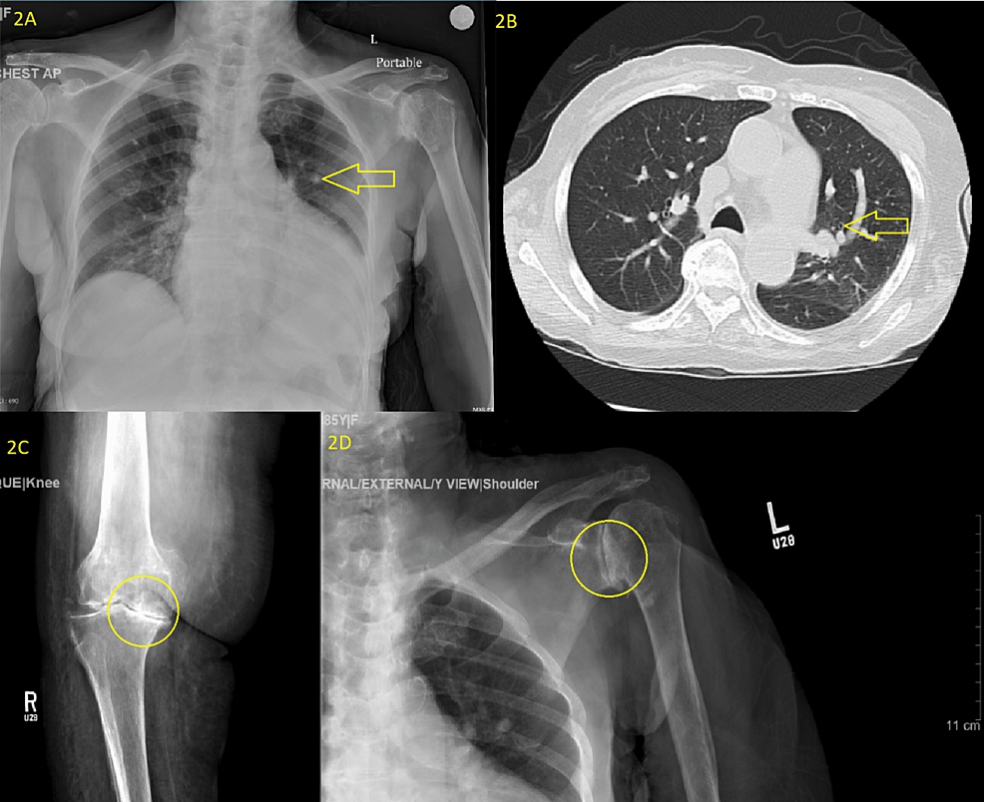
Diagnostic imaging on admission. A: Chest X-ray of acute pathology with a calcified granuloma (arrow). B: CT of the chest: centrilobular emphysema (arrow). Four-chamber cardiac enlargement and no lung mass, suspicious nodule, or active infiltrate or congestion. C: X-ray of the right knee showed severe osteoarthritis (circle). D: No acute pathology. Severe glenohumeral osteoarthritis (circle).

On admission, imaging (Figures 2A-2D) showed calcified granuloma, centrilobular emphysema, and severe arthritis of the knee and shoulder.

**Figure 3:**
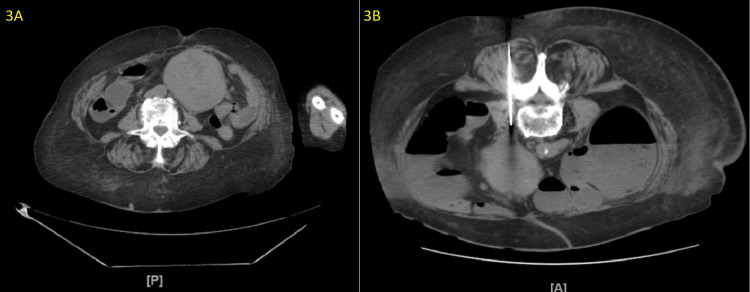
CT of the abdomen. A: CT of the abdomen and pelvis without contrast shows a 6.5 cm × 7.0 cm well-marginated mesenteric mass of the left paramedian abdomen. B: Biopsy of the mesenteric mass of the left paramedian abdomen without infiltration of the adjacent fat planes.

Computed tomography (CT) of the abdomen and pelvis without contrast (Figure 3A) done on admission showed a 6.5 cm × 7.0 cm well-marginated mesenteric mass of the left paramedian abdomen without infiltration of the adjacent fat planes. No other lesions were seen. Differential considerations included a mesenteric gastric tumor. Other etiologies could not be excluded. Follow-up was clinically advised. For her chronic persistent diarrhea, a stool workup was performed, including stool pH, fecal fat, white blood cells, culture (*Salmonella*, *Shigella*, *Campylobacter*, and *Escherichia coli*), osmolality, and *Clostridium difficile*. Stool culture, fecal fat, and *Clostridium difficile* were negative. The results showed stool of pH 7, no white blood cells, and osmolality of 363 mOsmol/kg, which was within the normal range. Hematology and oncology recommended consulting Interventional Radiology (IR). She underwent an IR-guided biopsy, as illustrated in Figure 3B.

**Figure 4:**
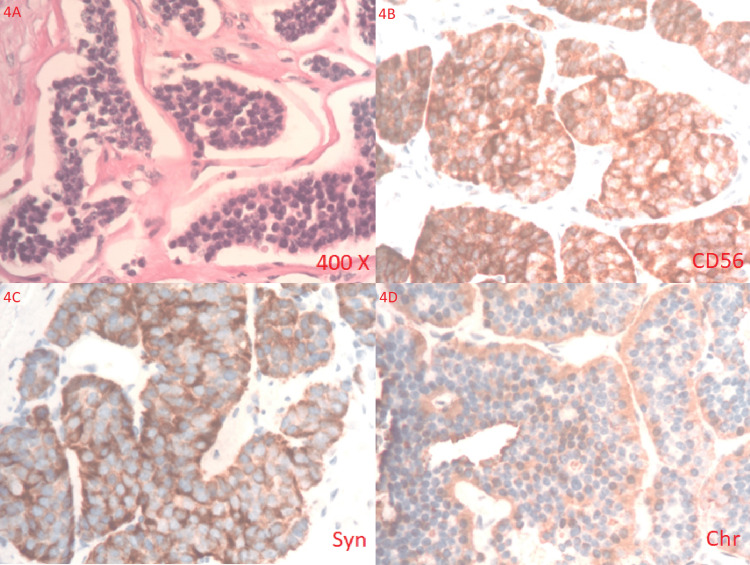
Histology of neuroendocrine tumor. A: A well-differentiated neuroendocrine tumor. The neuroendocrine tumor cells form various sizes of nests with monotonous cell populations with round, bland nuclei (400×). The neuroendocrine tumor cells express CD56 (B), synaptophysin (C), and chromogranin (D).

Tumor markers were as follows: carcinoembryonic antigen 6.4 ng/mL (slight elevation), cancer antigen 19-9 105 U/mL (slight elevation), and alpha-fetoprotein 5.8 ng/mL (normal). On the initial biopsy, the mesentery mass demonstrated nests of neuroendocrine (small cell)-type neoplasm; however, the findings were not immediately available which delayed the diagnosis. A plan was made for a positron emission tomography scan. Pathology was consulted for further staining to confirm the diagnosis, as shown in Figures 4A-4D.

**Figure 5:**
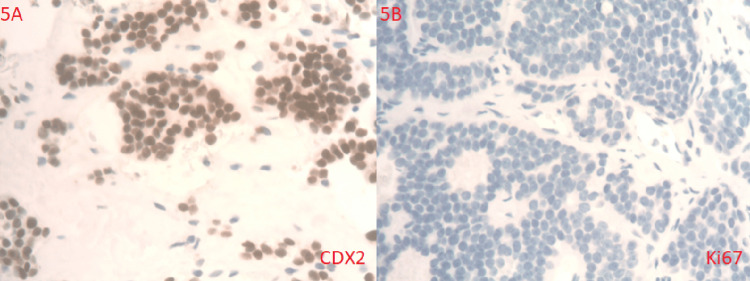
Immunoreactive histology of mesentery tumor cells. The mesentery tumor cells are immunoreactive to CDX2 (A) and Ki67 (B) is <1%.

Immunohistological staining was done to further confirm the NET as well as to assess for cellular proliferation (Figures 5A, 5B).

Before the biopsy, the patient’s diarrhea had resolved for four days but returned following the biopsy. She reported a recurrence of abdominal pain which was minimally relieved with NSAID. An abdominal X-ray showed dilated loops of bowel consistent with probable ileus, but no mechanical obstruction. The patient’s diet was changed to nothing by mouth with bowel rest per the recommendation of the surgical team. The following morning the patient presented with a recurrence of diarrhea and leukocytosis. The stool workup was again sent and the patient was started on antibiotics per the recommendation of infectious disease. The stool workup was repeated and the results were negative. The patient subsequently deteriorated and had to be intubated and placed on several vasopressors. At a later time, per the patient’s wishes, the family made the decision to compassionately extubate the patient and discontinue all vasopressor support.

## Discussion

Gastrointestinal neuroendocrine tumors (GI-NETs) account for nearly 70% of all carcinoid tumors. Approximately 25% of the remaining cases are located in the respiratory tract. GI-NETs are most common in the small intestine, rectum, appendix, or stomach [1]. Among the small intestinal segments, the ileum is the most frequently affected, with the duodenum and jejunum being the subsequent common sites [1]. Survival is highly dependent on the tumor stage. Gastric NETs account for 0.3% of all gastric tumors and 2% to 6% of all carcinoid tumors in the United States. Small intestine NETs constitute over half of all neoplasms in the small intestine [2]. In studies from Japan and other Asian countries, rectal NETs account for 60% to 90% of all GI-NETs [3,4]. Duodenal NETs comprise 5% of all GI-NETs [5].

While small intestine carcinoids are typically detected during the sixth decade of life [6], the diagnosis is frequently postponed by four to five years after the onset of symptoms, and there have been instances of even more extended delays [7]. Most (>90%) patients present with symptoms that are either related to tumor or to carcinoid syndrome. Tumor-related symptoms include abdominal pain, which is often intermittent and crampy in nature. Bowel obstructions can recur in cases, either as a result of the primary intestinal tumor or due to mesenteric metastases. Other signs/symptoms associated with these tumors include weight loss and hepatomegaly. Symptoms of carcinoid heart disease such as dyspnea and edema are often late manifestations of the disease [2]. Fewer NETs occur within the jejunum (9% to 18%) than within the ileum (70% to 87%), with 40% to 70% of the latter occurring within 2 feet of the ileocecal valve [1]. Small intestine NETs are generally small, with one-third being less than 1 cm, another third 1-2 cm, and the final third larger than 2 cm (with 8% >5 cm) [6].

On CT scan, mesenteric carcinoid tumors can display a range of characteristics, including different levels of fibrosis, calcification, and either focal or diffuse invasion of the neurovascular bundle by the tumor often involving both mechanisms [8]. NETs typically show positive results for specific immunohistochemistry markers such as synaptophysin, chromogranin A, cytokeratin, and CD-56 [9]. The histology report came back after our patient expired and showed synaptophysin, chromogranin A, and CD-56 were positive, confirming the diagnosis of NET cells from various sizes of nests with monotonous cell populations with round and bland nuclei. The tumor was positive for CDX2 but negative for CK7 and CK20. The Ki67 proliferative index of the well-differentiated NET is less than 1%, which means one in every 100 cells (1%) is dividing; therefore, a grade 1 NET (WD NET G1).

In African American patients, detecting the classic symptoms of carcinoid syndrome can be challenging, primarily because these symptoms may not be as readily apparent in individuals with darker skin tones. Furthermore, the initial clinical findings can often mimic other disease conditions, adding to the diagnostic complexity. In this case, the tumor’s unusual location further complicated the diagnostic process. Regrettably, the patient’s condition deteriorated rapidly before we could complete a comprehensive evaluation and initiate specific treatment for the NET. Ultimately, the family made the difficult decision to compassionately remove the patient from the mechanical ventilator. This case highlights the presence of an extremely rare large primary mesenteric NET, a condition not documented in the current literature.

## Conclusions

Soft tissue NETs may have an appearance on imaging studies that challenge physicians to make a correct diagnosis. Despite the rarity of these tumors, they should be included in the differential diagnoses of other gastrointestinal masses. Primary NETs are rare but serious illnesses with variable mortality and the exact mechanism remains obscure. In summary, this case report highlights an infrequently reported association between soft tissue primary NETs and the occurrence of no metastasis when diagnosed. Further studies can reveal the underlying mechanisms to aid in improved diagnosis and outcomes, as well as for the development of disease-specific treatment guidelines.
